# Assessing segmental versus non-segmental features in the ventral nervous system of onychophorans (velvet worms)

**DOI:** 10.1186/s12862-016-0853-3

**Published:** 2017-01-03

**Authors:** Christine Martin, Vladimir Gross, Hans-Joachim Pflüger, Paul A. Stevenson, Georg Mayer

**Affiliations:** 1 Department of Zoology, University of Kassel, Heinrich-Plett-Str. 40, D-34132 Kassel, Germany; 2 Institute of Biology, Neurobiology, Free University of Berlin, Königin-Luise-Str. 28-30, D-14195 Berlin, Germany; 3 Physiology of Animals and Behaviour, Institute of Biology, University of Leipzig, Talstraße 33, D-04103 Leipzig, Germany

**Keywords:** Segmentation, Nerve cord, Ganglia, Arthropod, Panarthropoda

## Abstract

**Background:**

Due to their phylogenetic position as one of the closest arthropod relatives, studies of the organisation of the nervous system in onychophorans play a key role for understanding the evolution of body segmentation in arthropods. Previous studies revealed that, in contrast to the arthropods, segmentally repeated ganglia are not present within the onychophoran ventral nerve cords, suggesting that segmentation is either reduced or might be incomplete in the onychophoran ventral nervous system.

**Results:**

To assess segmental versus non-segmental features in the ventral nervous system of onychophorans, we screened the nerve cords for various markers, including synapsin, serotonin, gamma-aminobutyric acid, RFamide, dopamine, tyramine and octopamine. In addition, we performed retrograde fills of serially repeated commissures and leg nerves to localise the position of neuronal somata supplying those. Our data revealed a mixture of segmental and non-segmental elements within the onychophoran nervous system.

**Conclusions:**

We suggest that the segmental ganglia of arthropods evolved by a gradual condensation of subsets of neurons either in the arthropod or the arthropod-tardigrade lineage. These findings are in line with the hypothesis of gradual evolution of segmentation in panarthropods and thus contradict a loss of ancestral segmentation within the onychophoran lineage.

**Electronic supplementary material:**

The online version of this article (doi:10.1186/s12862-016-0853-3) contains supplementary material, which is available to authorized users.

## Background

Currently, two competing hypotheses describe the phylogenetic position of onychophorans (velvet worms); they represent either the sister group of arthropods or the sister group of arthropods plus tardigrades [1–8]. Despite this ongoing debate, onychophorans play a key role for understanding the evolution of Panarthropoda (Onychophora + Tardigrada + Arthropoda) and accordingly are a broadly investigated animal group [9–25].

**Fig. 1:**
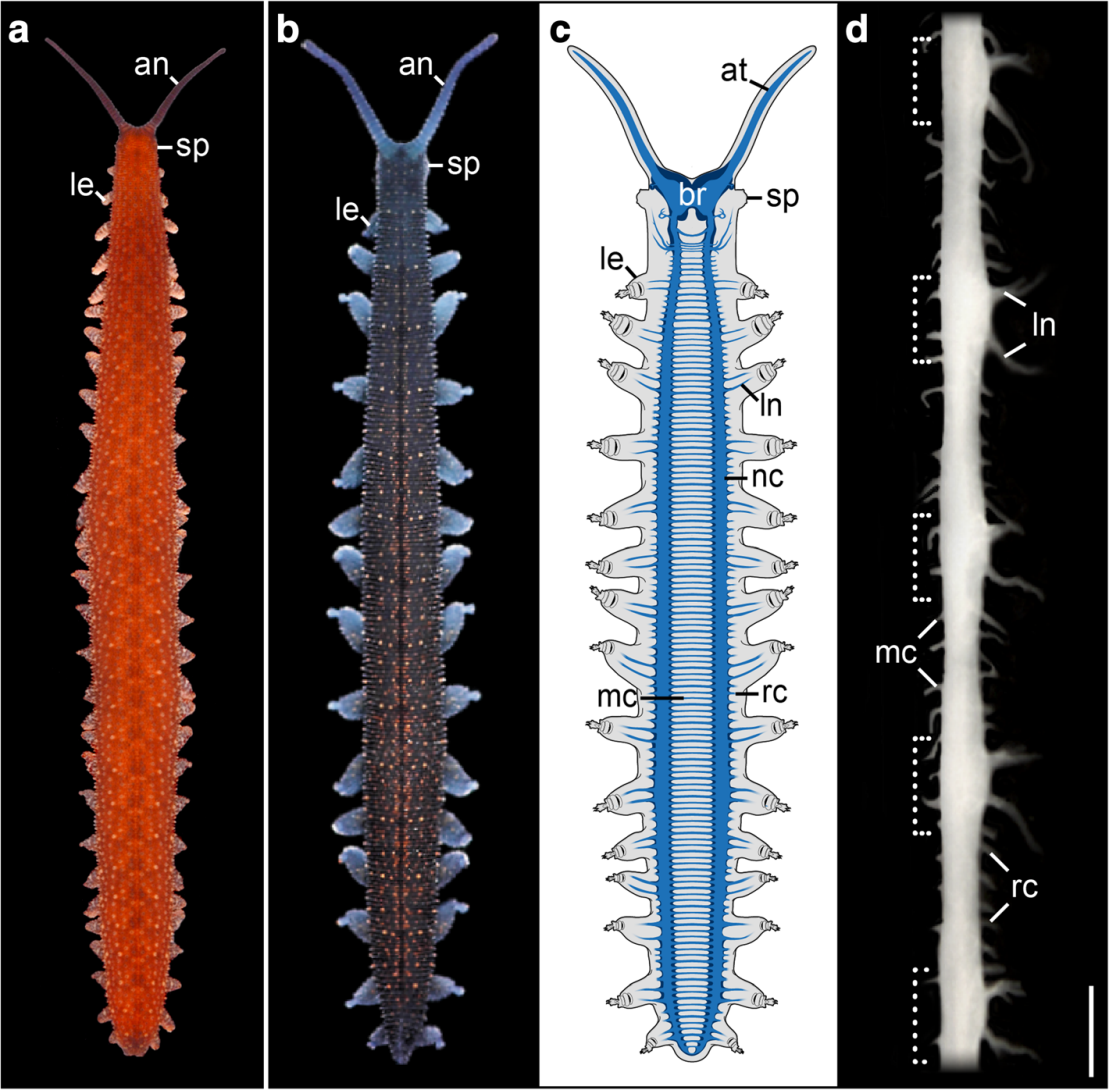
General anatomy and organisation of the central nervous system in Onychophora. Walking specimens of *Principapillatus hitoyensis* (**a**) and *Euperipatoides rowelli* (**b**). **c** Simplified diagram showing the entire onychophoran central nervous system with the brain situated within the head and the two ventral nerve cords located in the trunk. **d** Dissected single nerve cord from a specimen of *P. hitoyensis.* Anterior is up. Note the slight swelling in each leg-bearing region (*dotted brackets*). an, antenna; at, antennal tract; br, brain; le, leg; ln, leg nerve; nc, nerve cord; mc, median commissure; rc, ring commissure; sp, position of slime papilla. Scale bar: 50 μm (**d**)

One of the most enigmatic features of panarthropods is segmentation, which is evident in both arthropods and tardigrades, yet questionable in onychophorans. At first appearance, neither of the onychophoran groups (Peripatidae and Peripatopsidae) shows a conspicuous external segmentation (Fig. 1a, b), as clear boundaries of the cuticle are lacking [13]. The sole exception seems to be the appearance of segmentally repeated legs, although a closer look reveals additional segmental structures such as the ventral and preventral organs and crural papillae [26–28]. Internally, onychophorans also exhibit both segmental and non-segmental features. Whereas some structures such as pairs of leg nerves, nephridia, ostia of the heart and cellular strands associated with the midgut are segmentally repeated, other structures e.g. longitudinal musculature and ring and median commissures show no segmental arrangement [28–32]. Thus, the question arises of whether or not onychophorans are truly segmented animals or, more precisely, how much segmentation is actually present in onychophorans. To clarify this issue, it is important to define what a segment is and how segmentation evolved. To date, two competing interpretations of segmentation suggest that a segment either evolved as a holistic unit composed of at least six substructures [33, 34], which has been multiplied as a whole along the body, or that segmentation is simply the sum of individual repeated structures that resulted from concerted evolution (see [28, 35–37]). The latter hypothesis receives additional support from intermediate degrees of segmentation occurring in onychophorans [13, 28].

Comparative analyses of the nervous system are of particular use for clarifying such issues [38, 39]. One of the most intriguing aspects of the arthropod and tardigrade nervous systems is the segmental organisation of their ventral nerve cords [3, 40–42], which are subdivided into ganglia and somata-free connectives [39]. Several previous studies on the nervous system of onychophorans, specifically on the ventral nerve cords [11, 12], have shown that, in contrast to arthropods and tardigrades, onychophoran nerve cords lack clear segmental boundaries (Fig. 1c, d) even though some features are segmental while others are not. Whereas the only visible segmented structures are the repeated pairs of leg nerves, both ring and median commissures are arranged in a repeated but clearly non-segmental fashion as they show slight differences in number in each interpedal region even within the same specimen [12, 13, 43, 44]. Therefore, it is difficult to define onychophorans and their ventral nervous system as strictly segmented or non-segmented.

Nevertheless, due to thickenings within the leg-bearing regions of the onychophoran nerve cords, their nervous system was suggested to be either equipped with “anlagen” of segmental ganglia [45], “unfused ganglia” [16] or rudiments of ganglia (so-called “imperfect ganglia”, [9, 44, 46] but see [11] for an alternative view), thus implying that segmentally arranged ganglia were lost in the onychophoran lineage. This hypothesis has indeed been put forward previously [1, 47]. However, the thickenings correlate with the regions of the nerve cord that bear the laterally projecting leg nerves, which are noticeably thicker than the ring commissures (Fig. 1d) that also arise laterally, but originate only from the interpedal regions of the nerve cord. Therefore, we performed neuronal tracing experiments to compare numbers of somata supplying the leg nerves with those innervating the ring commissures in order to investigate a possible correlation of thickenings within the nerve cord with associated structures.

Furthermore, little is known about the adult onychophoran nerve cord in terms of the distribution of different neuronal markers (see [11, 12, 25]) and their segmental character. Quantitative analyses of these markers are completely lacking. Thus, we used a selection of established antibodies directed against known neurotransmitters and neuromodulators (serotonin, gamma-aminobutyric acid [GABA], dopamine, octopamine, tyramine, allatostatin, pigment-dispersing factor [PDF], referred to as pigment-dispersing hormone in crustaceans [48]; perisulfakinin, and RFamide) and a synaptic protein (synapsin) to review details of the internal organisation of key neuronal structures and their potentially segmental character (as revealed by statistical analyses) within the peripatopsid *Euperipatoides rowelli* (Fig. 1b), a model onychophoran. Finally, we discuss how the combination of segmental and non-segmental features in the onychophoran nerve cords relates to the evolution of segmental ganglia in Panarthropoda.

## Results

### General organisation of the onychophoran ventral nerve cord

**Fig. 2:**
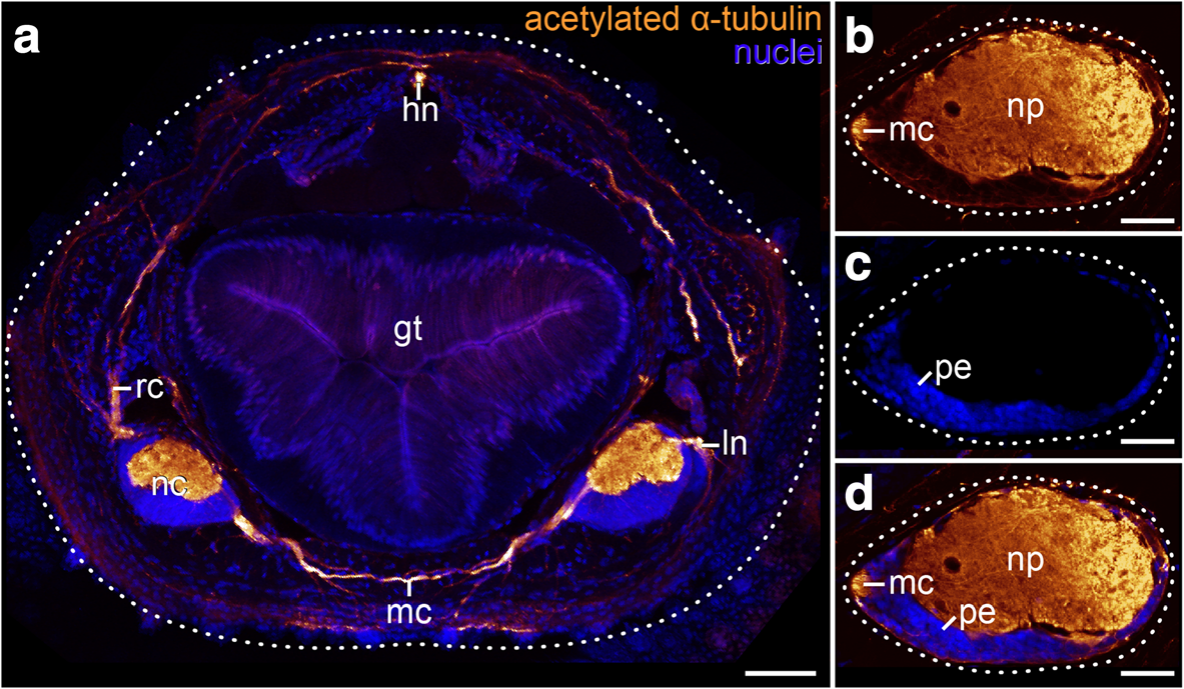
Organisation of the ventral nervous system in Onychophora as revealed by cross sections. **a**–**d** Maximum projections confocal micrographs of Vibratome sections of a specimen of *Euperipatoides rowelli* double-labelled with anti-acetylated α-tubulin serum (*glow mode*) and DNA marker Hoechst (bisbenzimide; *blue*). Dorsal is up in all images. Median is left (**b**–**d**). **a** Cross section through a whole specimen showing position of the ventral nerve cords that are connected via a median commissure (*mc*). **b**–**d** Cross section of a nerve cord. **b** Anti-acetylated α-tubulin labelling showing the neuropil. **c** DNA labelling with Hoechst showing the perikaryal layer. **d** Merged image of the same specimen as in (**b**) and (**c**). gt, gut; hn, heart nerve; ln, leg nerve; mc, median commissure; nc, nerve cord; np, neuropil; pe, perikaryal layer; rc, ring commissure. Scale bars: 100 μm (**a**), 50 μm (**b**–**d**)

**Fig. 3:**
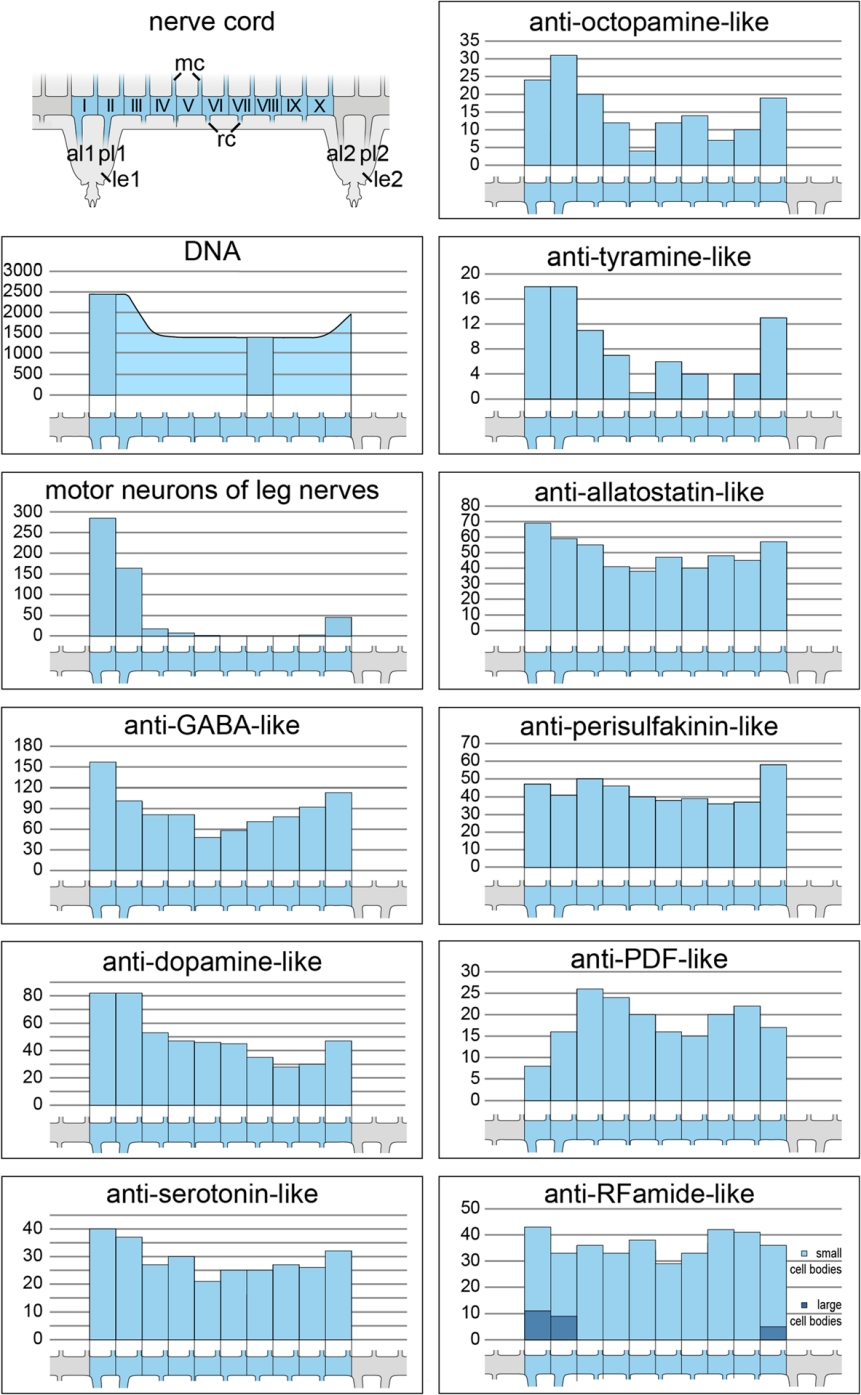
Estimated numbers of neuronal somata per hemisegment in Onychophora (*Euperipatoides rowelli*) as revealed by different markers. The upper left diagram shows the general organisation of a single ventral nerve cord in dorsal view (median is up) from one body side and two consecutive leg-bearing regions. To estimate the number of neuronal somata, the region of a nerve cord corresponding to a hemisegment (*light blue*) was subdivided into 10 equal regions (*I–X*), in which the somata were counted. Each hemisegment was defined by two consecutive anterior leg nerves (*al1* and *al2*). Bar graphs illustrate the counted numbers of labelled somata as identified by different antisera in corresponding regions of the nerve cord displayed below each diagram. Note that the number of cell bodies stained with a DNA-selective marker was estimated by using sections from the regions indicated by bar graphs. Anti-RFamide-like immunoreactive cell bodies were categorised in small (*light blue*) and large cell bodies (*dark blue*). Note also that only one specimen was analysed for each marker. al1 and al2, anterior leg nerve 1 and 2; le1 and le2, leg nerve 1 and 2; mc, median commissure; rc, ring commissure; pl1 and pl2, posterior leg nerve 1 and 2

The ventral nervous systems in both onychophoran species, the peripatopsid *Euperipatoides rowelli* and the peripatid *Principapillatus hitoyensis*, are characterised by two widely separated ventrolateral nerve cords that extend throughout the whole animal (Fig. 1c). The nerve cords are medially connected via numerous median commissures (Figs. 1c, d, 2a). Additionally, the two nerve cords are connected via numerous ring commissures, which run dorsolaterally along the body wall (Fig. 2a) but originate from a different level of the nerve cords than the median commissures. These ring commissures are restricted to the interpedal regions and hence are absent from the leg-bearing regions. Cross sections double stained with anti-acetylated α-tubulin serum and the DNA-marker Hoechst revealed a medullary structure of the ventral nerve cords, which are separated into a dorsomedial neuropil and a so-called perikaryal layer comprising ventromedial/ventral/ventrolateral cell bodies surrounding the neuropil, with the ventromedial portion of the perikaryal layer being the thickest (Fig. 2a–d). This composition can be detected consistently throughout the whole onychophoran nerve cords. For comparative analysis, we subdivided the nerve cord in hemisegments by using two consecutive posterior leg nerves as boundaries (Fig. 3 and Additional file 1A). To estimate the total number of neurons within one hemisegment of the ventral nerve cord, we counted the number of somata stained with the DNA-selective marker DAPI in a total of 16 sections (20 μm each) of one juvenile specimen of *Euperipatoides rowelli,* whereby seven sections were counted from the leg-bearing regions of the nerve cord and nine from the interpedal region (Additional file 1). Our analysis revealed a mean of 169.7 somata (standard deviation, SD 14.8) per section of the leg-bearing region and significantly fewer somata, i.e. a mean of 97.1 (SD 6.5), in the interpedal region (Table 1; *t* 13.28, df 14, *p* <0.001). Taking into account that the leg-bearing region is larger (mean area assuming a perfect ellipse = 20.94 μm^2^) than the interpedal region (mean area = 9.18 μm^2^) we calculated that the mean density of cells in the leg bearing regions (0.0082/μm^2^) is in fact approximately 23% less than in the interpedal regions (0.01059/μm^2^; t 4.737, df 10.53, *p* <0.001). Additionally, cross sections of the staining revealed different circumferences of the neuropil region between the interpedal (Additional file 1B) and the leg-bearing regions, (Additional file 1C) with the neuropil region being thicker within the leg-bearing region. By extrapolating the data from our sampled sections, and assuming equal distributions of somata, we estimated that the leg-bearing region of the nerve cord, having a length of approximately 702 μm (35 × 20 μm sections) contains ~5940 neurons in total, compared to the whole length of the interpedal region comprising ~2175 μm (109 × 20 μm sections), with an estimated total of 10,573 neurons. However, a length of the interpedal nerve cord equal to that of the leg-bearing region would contain ~3399 neurons and thus ~2540 neurons less than the total leg-bearing region. In total, DAPI revealed ~16,500 cell bodies within one hemisegment of the nerve cord in *E. rowelli* (Fig. 3 and Additional file 1B, C).

**Table 1:**
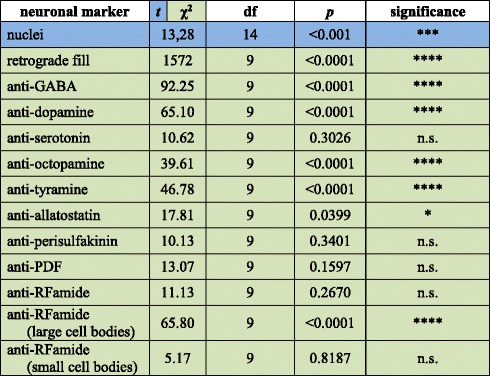
Results from Student’s *t*-test and χ^2^-tests for significant differences in the distribution of stained somata for each marker in *Euperipatoides rowelli*

The statistical significance for the Student’s *t*-test (*blue*) indicates that the deviation from the mean of stained somata within 20 μm thick sections of the leg-bearing region (seven sections) differs significantly (***, *p* < 0.001) from the deviation of the mean of stained somata within the interpedal region (nine sections). The statistical significance for the χ^2^-test (*green*; *, **, ***, ****, *p* < 0.05, 0.01, 0.001, 0.0001, respectively) indicates that the distribution of stained somata within the ten portions of one hemisegment differs significantly from a theoretically expected uniform distribution with no regional specialisation in the nerve cord. df, degrees of freedom for the test; n.s. not significant; *p*, probability of error

### Innervation pattern of peripheral nerves associated with the nerve cord

For our analyses we investigated the anatomy and composition of the onychophoran nerve cords, including their associated peripheral nerves, and estimated the number of supplying somata with neuronal tracing experiments (i.e. retrograde fills). For comparative analyses, we subdivided the nerve cords into hemisegments, which were in turn subdivided into ten equal portions using two consecutive anterior leg nerves as boundaries (see upper left diagram in Fig. 3).

**Fig. 4:**
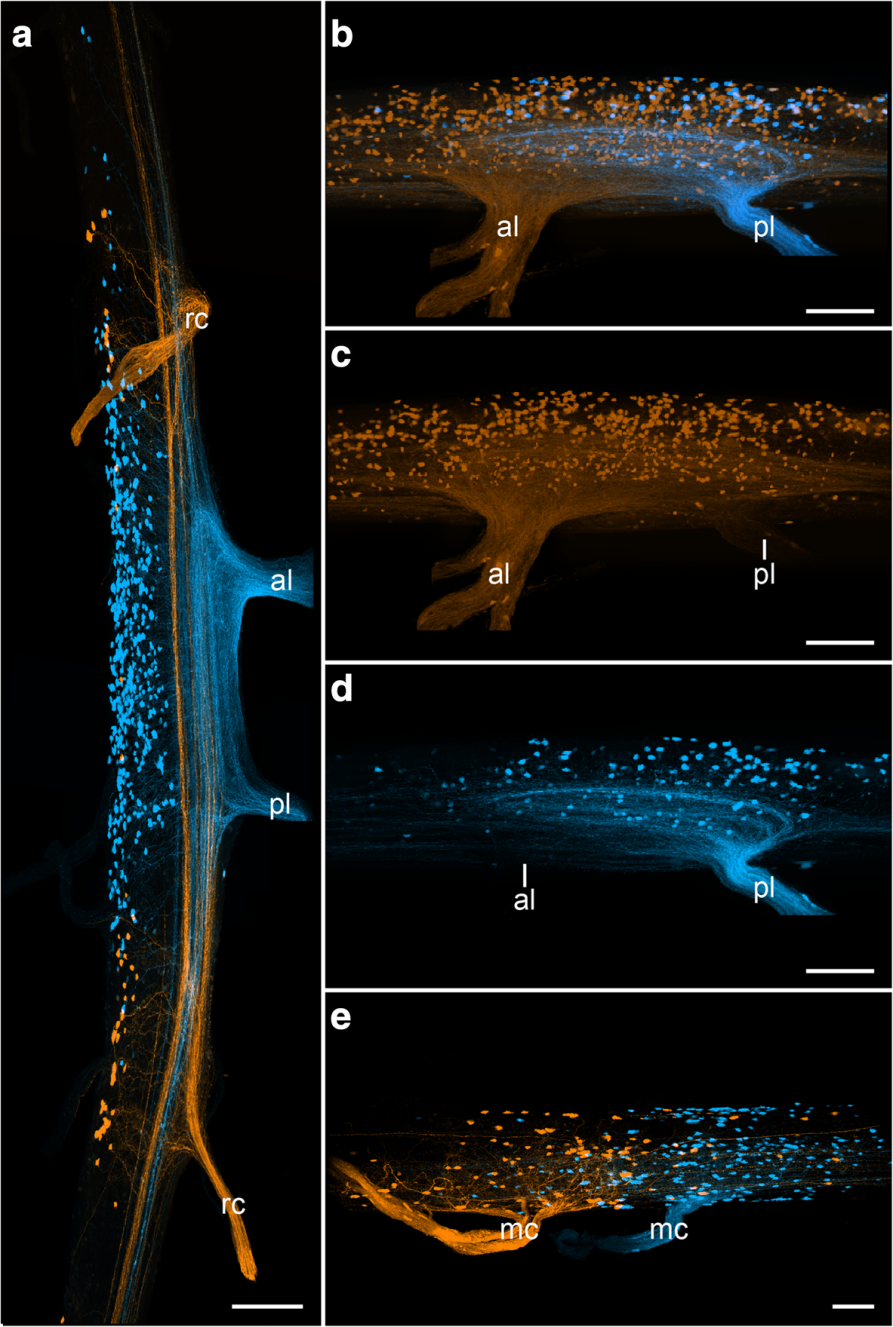
Retrograde tracing of nerves associated with the ventral nerve cords in Onychophora. Maximum projection confocal micrographs. Retrograde fills with dextran coupled to either tetramethylrhodamine or fluorescein. Anterior is up (**a**) or left (**b**–**d**). Median is left (**a**)**,** up (**b**–**d**) or down (**e**). **a** Position of neuronal cell bodies associated with ring commissures, and the anterior and posterior leg nerves in *Euperipatoides rowelli*. **b**–**d** Position of motor neurons associated with the anterior and posterior leg nerves in *E. rowelli* (**b**) and a separated depiction of the anterior (**c**) and the posterior leg nerves (**d**) of the same labelling as in **b. e** Position of somata associated with two subsequent median commissures in *Principapillatus hitoyensis.* al, anterior leg nerve; mc, median commissure; rc, ring commissure; pl, posterior leg nerve*.* Scale bars: 100 μm (**a**) and 50 μm (**b**–**d**)

**Fig. 5:**
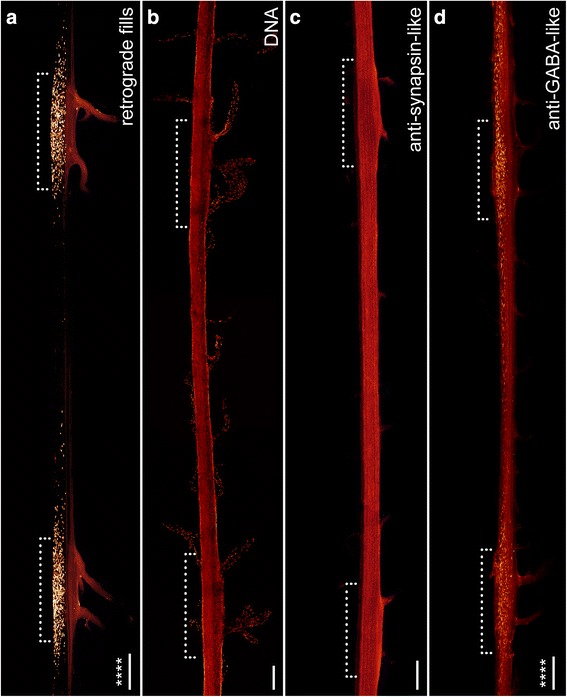
Organisation of individual ventral nerve cords in Onychophora as revealed by different neuronal markers: retrograde fills (**a**), DNA labelling (**b**), antiserum against synapsin (**c**) and GABA (**d**). Maximum projection confocal micrographs of *Euperipatoides rowelli*. Median is left. *Asterisks* indicate that the distribution of somata is significantly different from uniform (χ^2^ *, **, ***, ****, *p* < 0.05, 0.01, 0.001, 0.0001 respectively; n.s. not significant; see Table 1 for more details). *Dotted brackets* indicate leg-bearing regions characterised by the position of leg nerves. **a** Note that the somata of neurons associated with leg nerves are grouped near their bases (original data from Mayer et al. [3], creative common license of BMC Evol Biol). **d** Note also the condensed groups of neuronal cell bodies labelled against the neuropeptide GABA that are also located near the bases of leg nerves. Scale bars: 200 μm

The ventral nerve cords are associated laterally with both leg nerves and ring commissures (Figs. 1d, 2a, 4a–d). Each leg is innervated by two nerves, the anterior and the posterior leg nerve, which can easily be distinguished from the ring commissures by their more substantial appearance (Figs. 4a, 5a). Both leg nerves of each leg ramify (Fig. 4b–d) but differ in that the anterior leg nerve branches more proximally (Fig. 4b, c). Nevertheless, our neuronal tracing experiments revealed a similar innervation pattern of both leg nerves supplying one leg (Figs. 4a–d, 5a). The cell bodies of motor neurons supplying the leg nerves are distributed within the nerve cord at the same level longitudinally but on the medial side, within the perikaryal layer (Figs. 4c, 5a). Furthermore, the cell bodies of these motor neurons do not differ appreciably in size, being all ~8 μm in diameter. Separate retrograde tracing experiments of the anterior and posterior leg nerve revealed a mirrored pattern of the position of motor neurons supplying each of the two nerves of one leg (Fig. 4b–d). Somata and neuronal fibres of both the anterior and the posterior leg nerve are concentrated towards the midpoint of a leg-bearing region. Thus, neurons supplying the anterior leg nerve show a posterior shift (Fig. 4b, c) whereas neurons supplying the posterior leg nerve are shifted anteriorly (Fig. 4b, d) with respect to the nerve itself. In total, one hemisegment of the nerve cord comprises ~530 cell bodies of motor neurons innervating both leg nerves (Fig. 3), almost all of which are concentrated within the leg-bearing regions (Table 1; χ^2^ 1572, df 9, *p* <0.0001; Figs. 4 a–d, 5a).

In addition, we performed neuronal tracing experiments on the ring commissures, which also arise laterally from the onychophoran nerve cords (Figs. 2a, 4a). Similar to the leg nerves, the somata occur within the nerve cord on the opposite side of each commissure (Fig. 4a). However, the neuronal cell bodies of the ring commissures do not show an anterior or posterior shift in position in comparison to the commissure, but instead are evenly distributed at the same level as the commissure. In total, we estimated ~40 neuronal cell bodies innervating one ring commissure. In contrast to the somata of the leg nerves, those supplying these commissures differ in size (Fig. 4a). The majority of cell bodies are ~8 μm in diameter but some are almost double the size (up to 16 μm).

Furthermore, retrograde fills of the median commissures, which connect the two nerve cords ventrally (Figs. 3a, 4e), revealed that their somata are distributed ventro-laterally in the nerve cord at the same level as each respective commissure (Fig. 4e; revealed in *Principapillatus hitoyensis*). We estimated a number of approximately 130 somata supplying one median commissure. In contrast to the somata supplying the leg nerves, an anterior or posterior shift in position is not evident. Moreover, the neuronal cell bodies of median commissures do not show any differences in size (all are ~11 μm in diameter) as seen in ring commissures.

### Subdivision of the onychophoran nerve cord into a perikaryal layer and a synaptic neuropil

**Fig. 6:**
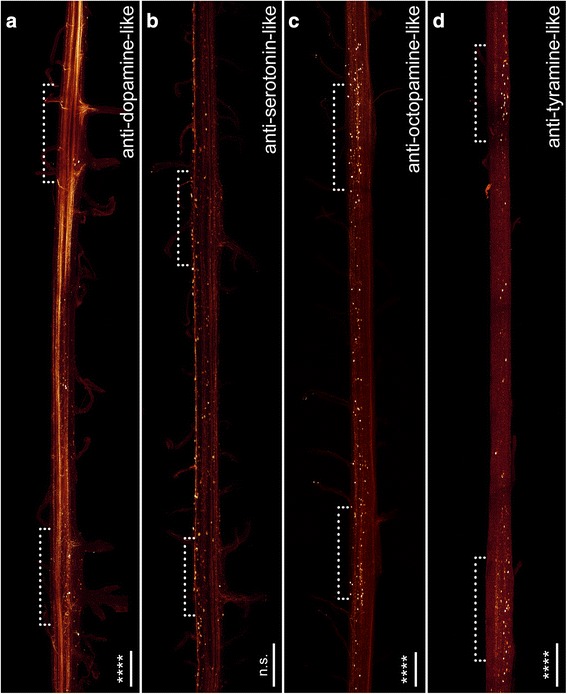
Organisation of individual ventral nerve cords in Onychophora as revealed by different neuronal markers: antisera against dopamine (**a**), serotonin (**b**), octopamine (**c**) and tyramine (**d**). Maximum projection confocal micrographs of *Euperipatoides rowelli* (**c**). Median is left. *Asterisks* indicate the distribution of somata that is significantly different from uniform (χ^2^ *, **, ***, ****, *p* < 0.05, 0.01, 0.001, 0.0001 respectively; n.s. not significant; see Table 1 for more details). *Dotted brackets* indicate leg-bearing regions characterised by the position of leg nerves. **d** Note the condensed groups of neuronal cell bodies labelled against the amine tyramine that are present near the bases of leg nerves. Scale bars: 200 μm

**Fig. 7:**
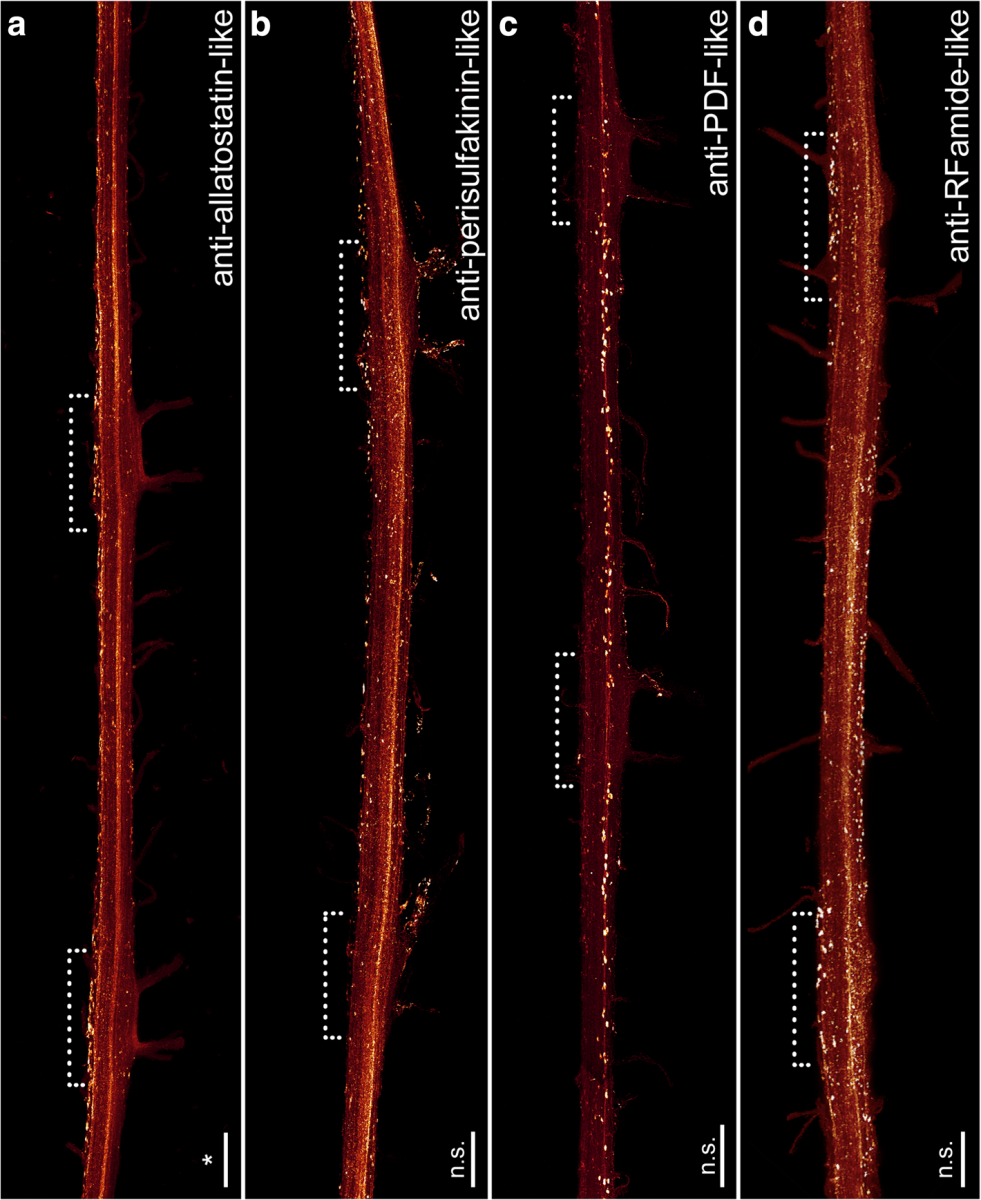
Organisation of individual ventral nerve cords in Onychophora as revealed by different neuronal markers: antisera against allatostatin (**a**), perisulfakinin (**b**), pigment-dispersing-factor (PDF; **c**) and RFamide (**d**). Maximum projection confocal micrographs of *Euperipatoides rowelli*. Median is left. *Asterisks* indicate the distribution of somata that is significantly different from uniform (χ^2^ *, **, ***, ****, *p* < 0.05, 0.01, 0.001, 0.0001 respectively; n.s. not significant; see Table 1 for more details). *Dotted brackets* indicate leg-bearing regions characterised by the position of leg nerves. **d** Note the condensed groups of neuronal cell bodies labelled against the peptide RFamide that are present near the bases of leg nerves. Scale bars: 200 μm

**Fig. 8:**
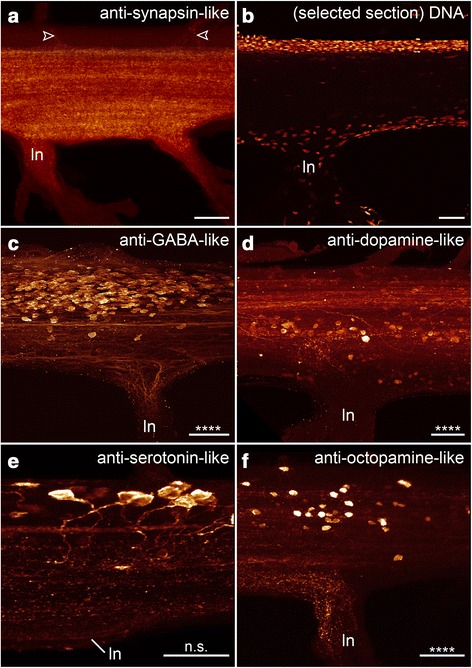
Detailed organisation of the ventral nerve cords Onychophora as revealed by different markers: antiserum against sysnapsin (**a**), DNA labelling (**b**), antisera against GABA (**c**), dopamine (**d**), serotonin (**e**) and octopamine (**f**). Maximum projection confocal micrographs of *Euperipatoides rowelli* at the level of leg nerve. Median is up in all images. *Asterisks* indicate the distribution of somata that is significantly different from uniform (χ^2^ *, **, ***, ****, *p* < 0.05, 0.01, 0.001, 0.0001 respectively; n.s. not significant; see Table 1 for more details). **a** *Arrowheads* point to few immunoreactive fibres projecting through the perikaryal layer. **b** Note that the DNA labelling shows a detailed organisation only of a thin sagittal section of the middle part of the nerve cord, hence not all nuclei are visible. ln, leg nerve. Scale bars: 50 μm

**Fig. 9:**
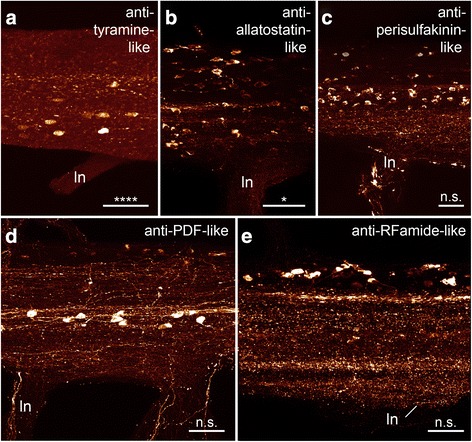
Detailed organisation of the ventral nerve cords in Onychophora as revealed by different markers: antisera against tyramine (**a**), allatostatin (**b**), perisulfakinin (**c**), pigment-dispersing-factor (PDF; **d**) and RFamide (**e**). Maximum projection confocal micrographs of *Euperipatoides rowelli* at the level of the leg. Median is up. Cases where the distribution of somata is significantly different to uniform are indicated (χ^2^ *, **, ***, ****, *p* < 0.05, 0.01, 0.001, 0.0001 respectively; n.s. not significant; see Table 1 for more details). ln, leg nerve. Scale bars: 50 μm

A variety of neuronal markers were employed to obtain a more detailed insight into the composition of the nerve cord (Figs. 5, 6 and 7 for an overview and Figs. 8, 9 for a detailed view) in *Euperipatoides rowelli*. A DNA-selective fluorescent dye revealed that the entire complement of neuronal cell bodies is distributed uniformly along the periphery of each nerve cord (Figs. 2a, c, d, 5b). Cross and sagittal sections revealed a relatively thick layer of cell bodies in the medial part and a thinner layer of somata in the lateral part of the nerve cords (Figs. 2a, c, d 8b). No cell bodies were found in the interior region, which instead composes the synaptic neuropil, as revealed by an antiserum against the vesicle-associated protein synapsin (Figs. 5c, 8a). This synaptic neuropil mass extends uniformly throughout the whole nerve cord. In contrast, the medial perikaryal layer is largely free of synapsin-like immunoreactivity (synapsin-LIR) except for few immunoreactive neurites that project through the perikaryal layer to the median commissures (arrowheads in Fig. 8a). Additionally, synapsin-like immunoreactive profiles extend to both laterally located ring commissures and leg nerves (Figs. 5c, 8a). Particularly, the base of the leg nerves shows a strong synapsin-LIR that decreases peripherally. Both DNA-labelling and labelling with an anti-synapsin serum revealed slight thickenings of the nerve cords within the leg-bearing regions compared to the interpedal regions of the same nerve cord. Those thickenings differ in size for each investigated nerve cord and range from 110 to 130% of the adjacent interpedal regions in both groups investigated.

### Immunoreactivity within the onychophoran nerve cord as revealed by antisera against different neurotransmitters and neuromodulators

For a more detailed understanding of the composition of nerve cords in onychophorans we performed immunohistochemical staining with different antisera against several neurotransmitters and neuromodulators to reveal the distribution of immunoreactive neuronal cell bodies (see Figs. 5d, 6, 7 for an overview and Figs. 8, 9 for a detailed view) and estimated the number of immunoreactive somata labelled with each specific marker per hemisegment for comparative analysis (Fig. 3) within the model onychophoran *Euperipatoides rowelli*.

Our investigations revealed a relatively high number of GABA-like immunoreactive (GABA-LIR) cell bodies along the whole nerve cord whereby an increased number occurs in the leg-bearing regions (Fig. 5d). The uniformly sized cell bodies are mainly distributed in the medial perikaryal layer opposite to the leg nerves (Fig. 8c). In total, ~880 GABA-LIR neuronal cells bodies occur within one hemisegment of the nerve cord (Fig. 3), with almost double that number within a section of the leg-bearing region compared to a section of an interpedal region of the nerve cord (Table 1; χ^2^ 92.25, df 9, *p* <0.0001; Fig. 5d). In general, GABA-LIR somata are more numerous than those labelled by any other immunohistochemical marker investigated herein (Fig. 3). A slightly weaker GABA-LIR signal is detected within neurites running medially through the nerve cord, some of which project towards the leg nerves, where the signal decreases distally.

Immunohistochemical investigations with an antiserum against the neurotransmitter dopamine revealed a strong immunoreactivity of the neuropil fibres (Fig. 6a). Dopamine-LIR neurites are also present within the leg nerves and median commissures (Fig. 6a). Furthermore, dopamine-LIR cell bodies occur within the perikaryal layer (Figs. 6a, 8d), and are significantly more numerous in the regions that innervate the leg nerves (Table 1; χ^2^ 65.10, df 9, *p* <0.0001; Figs. 3, 6a). In total, immunohistochemistry revealed ~495 dopamine-LIR neuronal cell bodies within one hemisegment of the nerve cord. Furthermore, many fibres show a strong dopamine-LIR running through the median neuropil within the nerve cord (Figs. 6a, 8d). Additionally, several of the dopamine-LIR neurites project towards the base of the leg nerves (Fig. 6a).

Serotonin-LIR neuronal cell bodies are distributed along the whole nerve cord (Fig. 6b), with a slightly higher number occurring within the leg-bearing regions (Fig. 3). However, this pattern is less evident as e.g. with GABA-LIR or dopamine-LIR somata and appears to be not significant (Table 1; χ^2^ 10.62, df 9, *p* 0.3026). In total, we estimated ~290 serotonin-LIR cell bodies within one hemisegment (Fig. 3), all of which are relatively larger in size (~18 μm) compared to all other immunoreactive somata investigated (Fig. 8e). Additionally, the unipolar cell bodies of serotonin-LIR neurons exhibit prominently labelled axons projecting from the perikaryal layer to the synaptic neuropil mass (Fig. 8e).

The distribution pattern of neuronal cell bodies showing octopamine-LIR (Fig. 6c) is similar to that obtained with antisera against the neurotransmitters GABA and dopamine, i.e. immunoreactive cell bodies are distributed along the whole nerve cord but with a significantly higher number within the leg-bearing regions (Table 1; χ^2^ 39.61, df 9, *p* <0.0001; Figs. 3, 6c). Our investigations revealed a total number of ~150 octopamine-LIR somata within one hemisegment in the nerve cord, all of which are located within the medial perikaryal layer (Figs. 6c, 8f). Furthermore, octopamine-LIR can be seen within the neuropil and within single neurites projecting to the median and ring commissures (Figs. 6c, 8f). The fibres within the base of the leg nerves especially show a prominent immunoreactive signal that decreases peripherally (Fig. 8f).

A similar distribution pattern is seen using an antiserum against tyramine (Figs. 6d, 9a). Here, the number of immunoreactive cell bodies is, with ~80 per hemisegment, the lowest of all neurotransmitters investigated (Fig. 3). Nevertheless, the occurrence of a significantly larger number of immunoreactive somata within the leg-bearing regions becomes clear, as some parts of the interpedal regions contain no immunoreactive somata (Table 1; χ^2^ 46.78, df 9, *p* <0.0001; Figs. 3, 6d).

Cell bodies labelled with an antiserum against the neuropeptide allatostatin show a distribution pattern similar, albeit less evident, to most of the neurotransmitters in the onychophoran nerve cord (Fig. 7a). The allatostatin- LIR cell bodies are distributed along the whole nerve cord, but with significantly more within the leg-bearing regions (Table 1; χ^2^ 17.81, df 9, *p* 0.0399; Figs. 3, 7a). In total, one hemisegment comprises ~500 allatostatin-LIR somata in the nerve cord (Fig. 3). These occur within the medial as well as the lateral perikaryal layer (Figs. 7a, 9b). Additionally, allatostatin-LIR is present within the neuropil, where a thick fibre bundle, running centrally within the nerve cord, shows strong immunoreactivity.

A specific distribution pattern is not visible for perisulfakinin-LIR neuronal cell bodies, as the somata are distributed rather indiscriminately along the whole nerve cord (Table 1; χ^2^ 10.13, df 9, *p* 0.3401; Figs. 3, 7b) within both lateral and medial perikaryal layers (Fig. 9c). One hemisegment of the nerve cord comprises ~430 perisulfakinin-LIR somata (Fig. 3). Moreover, the fibres within the neuropil and a few fibres projecting to leg nerves, ring and medial commissures also exhibit a prominent perisulfakinin-LIR.

Our immunohistochemical investigations with an antiserum against the neuropeptide pigment-dispersing factor (PDF) revealed labelled cell bodies distributed along the whole nerve cord but without any variation in any specific regions (Table 1; χ^2^ 13.07, df 9, *p* 0.1597; Figs. 3, 7c). Noticeably, the antiserum against PDF exhibits two different sizes of cell bodies: small cell bodies (~8 μm) distributed along the medial perikaryal layer and relatively large cell bodies (~14 μm) distributed ventromedially along the neuropil of the onychophoran nerve cord (Figs. 7c, 9d). Approximately 200 PDF-like immunoreactive somata are present within one hemisegment of the nerve cord (Fig. 3). Furthermore, the neuropil exhibits a bundle of neurites that shows a strong PDF-LIR at the same level as the large PDF-LIR somata (Fig. 9d). Additionally, some few PDF-LIR fibres project into the leg nerves and both ring and median commissures.

The RFamide-like immunoreactivity shows a unique distribution pattern of neuronal cell bodies. The majority of RFamide-LIR somata (~8 μm) is distributed laterally within the perikaryal layer of the nerve cord (Fig. 7d). Additionally, some larger somata (~16 μm) occur within the median perikaryal layer but solely within the leg-bearing region (Figs. 7d, 9e). However, this variation within the leg-bearing region compared to the interpedal region does not appear to be significant (Table 1; χ^2^ 11.13, df 9, *p* 0.2670), if taking all RFamide-LIR somata into account. When considered separately, at least the larger cell bodies show a highly significant arrangement, with all cell bodies occurring within the leg-bearing region (Table 1; χ^2^ 65.80, df 9, *p* <0.0001), whereas the small RFamide-LIR cell bodies are distributed without any significant differences in their arrangement along the nerve cord (Table 1; χ^2^ 5.176, df 9, *p* 0.8187). We calculated ~390 RFamide-like immunoreactive somata within one hemisegment in the onychophoran nerve cord (Fig. 3) distributed within the medial and lateral perikaryal layers. Additionally, prominently labelled neurites occur within the neuropil (Fig. 7d, 9e).

## Discussion

### Segmental and non-segmental features of the onychophoran nerve cord

Previously, several researchers suggested the onychophoran nervous system to be segmentally structured due to thickenings within leg-bearing regions [9, 16, 45]. Our data confirm the existence of swellings within the leg-bearing regions, which seem to show considerable differences between the onychophoran subgroups (Peripatidae versus Peripatopsidae), between specimens within the same group and even between hemisegments within the same specimen. The impression of these thickenings might be enhanced due to a wavy appearance of the nerve cords within the animal, with the outwardly protruding regions corresponding to the position of the leg nerves [29]. Additionally, this pattern is exaggerated in dissected nerve cords due to dissection artefacts resulting from either compression or stretching during treatment. Nevertheless, a DNA-selective marker not only revealed a higher number of cell bodies occurring within the region of the thickenings (i.e. leg-bearing regions) but also a larger size of the nerve cord in general (including a larger neuropil region) within these regions. Furthermore, a higher concentration of cell bodies occurring within the leg-bearing region could not be detected as the larger number of somata correlates with the larger size of the nerve cord within these regions. As the thickenings occur within the regions of the nerve cord that bear the laterally projecting, relative thick leg nerves, we suggest a correlation between thickenings and associated structures. In fact, our neuronal tracing experiments of the leg nerves revealed a significantly higher number of neuronal cell bodies that occur almost exclusively within the thicker leg-bearing regions than somata of the ring commissures, which are located within the interpedal regions. Hence, the swellings occurring within the regions bearing the leg nerves are attributable to a high number of neurons within these regions compared to the interpedal regions.

**Fig. 10:**
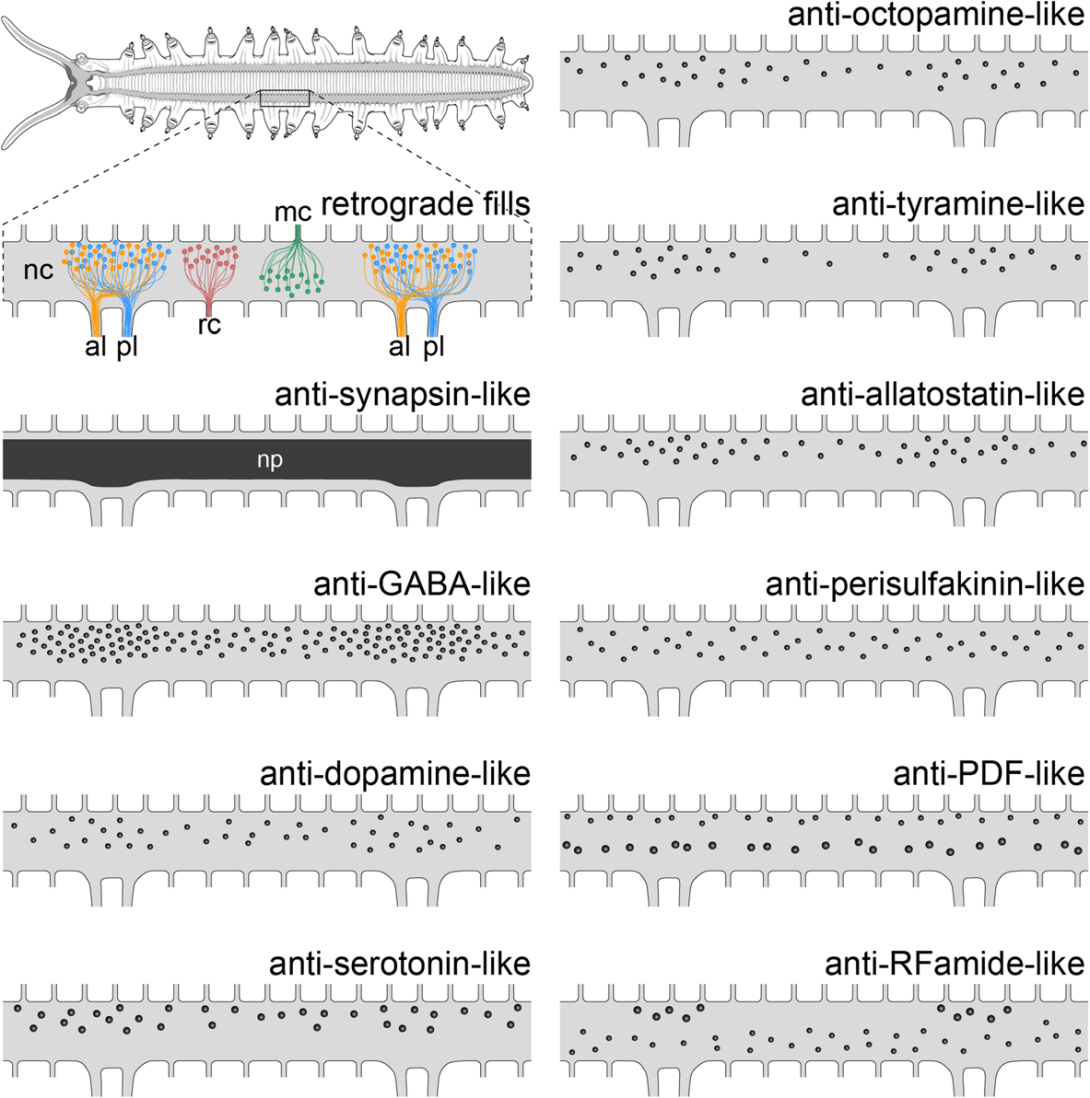
Simplified diagrams of onychophoran nerve cords summarising the results of retrograde fills and immunohistochemistry. The *upper left diagram* shows the entire central nervous system in dorsal view (anterior is *left*). The remaining diagrams illustrate detailed nerve cords from one body side of two consecutive leg-bearing regions. al, anterior leg nerve; mc, median commissure; nc, nerve cord; pl, posterior leg nerve; rc, ring commissure

Additionally, our investigations with different antibody labelling revealed two major distribution patterns of neuronal cell bodies — either the immunoreactive somata are broadly distributed along the whole nerve cord (as seen e.g. for anti-perisulfakinin or anti-PDF labelling) or the neuronal somata show an increased density within the segmentally repeated leg-bearing regions (Fig. 10). Furthermore, some markers, e.g. dopamine, allatostatin, and perisulfakinin, additionally show strongly labelled fibres. GABA-LIR is one example where the strongest signal is found in the leg-bearing regions (Fig. 10), including some neurites in the leg nerves, suggesting a role in innervation of the leg musculature. This locomotory function corresponds with other animal groups, where GABA-LIR is associated with body wall musculature in annelids [49] and leg muscles in arthropods [50]. Additionally, our results show that GABA-LIR is represented by the largest number of neurons of any marker that we tested in the onychophoran nerve cord, similar to what was found both in the earthworm *Lumbricus terrestris* (8% of total nerve cells [49]) and in the cricket *Gryllus bimaculatus* (up to 20% of ganglionic cells [51]).

Immunolabelling against dopamine, another marker that is concentrated in the leg-bearing regions (Fig. 10), also revealed similarities to previous results from arthropods. Similar to the moth *Manduca sexta*, where dopamine in the thoracic ganglia influences flight motor patterns [52], the concentration of dopamine-LIR cell bodies in the leg-bearing regions of onychophorans suggests that this signalling molecule may be involved in locomotion in these animals as well. On the other hand, the strong signal seen from dopamine-LIR fibres in the onychophoran nerve cord matches that of the cricket, where dopamine-LIR is prominent in segment-spanning central interneurons [53].

In the peripatopsid *E. rowelli*, RFamide-LIR cell bodies are distributed in a unique fashion, with the majority of cell bodies spread equally along the nerve cord but with some larger somata occurring only within the leg-bearing region. In arthropods, RFamide is known to have an impact on a variety of functions such as regulation of the circulatory system and modulation of the digestive tract and somatic muscles [54, 55]. The occurrence of the larger RFamide-LIR somata within the regions of the legs in onychophorans leads to the assumption that they might have an influence on neurons supplying the leg musculature.

Finally, anti-octopamine immunolabelling similarly shows increased immunoreactivity around the area of the leg nerves. This arrangement observed in the onychophoran ventral nerve cord may be related to the segmentally arranged, bilaterally paired, octopaminergic neurons of most arthropods [56]; however, insects are unique in that they possess unpaired dorsal and ventral median neurons (DUM and VUM neurons, respectively). With these special cells in mind, a pattern emerges across the arthropods with octopaminergic neurons being concentrated towards the midline of the body. This is evident not only in the DUM and VUM neurons of insects, but also in the paired octopaminergic neurons in the spider *Cupiennius salei* [57] and the lobster *Homarus americanus* [58, 59]. Our results reveal a similar pattern, with octopamine-LIR cells localising mainly in the medial half of the nerve cord, with octopamine-LIR even being associated with the median and ring commissures. In line with arthropods, the number of octopamine-LIR cells is relatively low compared to the other markers. On the other hand, octopamine in the arthropod central nervous system is present in a higher number of neuronal tracts, a distribution that is marginally observed in the onychophoran nerve cords.

Interestingly, the onychophoran musculature, like the nervous system, is composed of both segmental and asegmental elements. The body wall musculature does occur in a repeated pattern but is not segmentally arranged, whereas the musculature of the limbs appears in a segmental pattern [26, 30]. This pattern is reflected by the nervous system, where the repeated occurrence of two leg nerves supplying each leg and its associated motor neurons appear to be the only segmental pattern of the otherwise asegmental nerve cord in onychophorans [11, 12, 21]. The ring commissures, which also arise laterally and are repeated, cannot be considered segmental structures, as they occur at slightly different positions and vary in number in each body segment even within the same specimen [12, 13, 29]. These dorsolateral, non-segmental, repeated commissures supply the asegmental body wall musculature [29]. Thus, the segmental arrangement, or lack thereof, of neuronal cell bodies within the onychophoran nerve cord is more likely a product of the segmental nature of the structures they supply.

### Evolution of segmental ganglia in panarthropods

Our investigations revealed both segmental and non-segmental arrangements of neurons within the onychophoran ventral nerve cord (Fig. 10). While such indications have already been observed previously, the segmental arrangement of leg nerves and slight swellings within the nerve cord in these regions led to the assumption of a loss of segmental ganglia, which are present in arthropods and tardigrades, within the onychophoran lineage [9, 45, 60]. Thus, segmental ganglia were said to be an ancestral feature of panarthropods ([1] but see [7, 11–13, 17, 21, 25, 61]). According to the definition of Richter et al. [39], ganglia are an accumulation of cell bodies connected via somata-free connectives. Therefore, a loss of ganglia in the strict sense would result in a complete loss of cell bodies within the onychophoran ventral nervous system. Instead, the onychophoran ventral nervous system in this case would rather have experienced a re-distribution of cell bodies that were previously located within ganglia.

Nevertheless, our data clearly show a mixture of segmentally repeated and non-segmentally arranged cell bodies labelled with several antibodies. Additionally, the somata supplying the legs also occur in a segmental pattern at the base of each pair of leg nerves but they cannot be interpreted as remnants of ganglia, as the position of motor neurons is not comparable to a whole ganglion of arthropods, of which motor neurons are just a subset (see definition of ganglia in Richter et al. [39]). Instead, the arrangement of cell bodies corresponds to the arrangement of both segmental and non-segmental regions of the musculature or peripheral nervous system. Consequently, we find no evidence that these cell bodies were ever condensed into ganglia in the ancestor and subsequently redistributed in the onychophoran lineage.

However, the absence of segmentally arranged ganglia does not automatically imply an asegmental ventral nervous system in onychophorans. Hence, the issue is not whether or not the onychophoran nervous system is segmental but rather how much segmentation is present within their nerve cords and, moreover, whether or not the presence of segmentation is an ancestral feature of panarthropods. Unfortunately the phylogenetic position of onychophorans and tardigrades is still under debate, as either tardigrades or onychophorans or both form the sister group of arthropods [1–8].

**Fig. 11:**
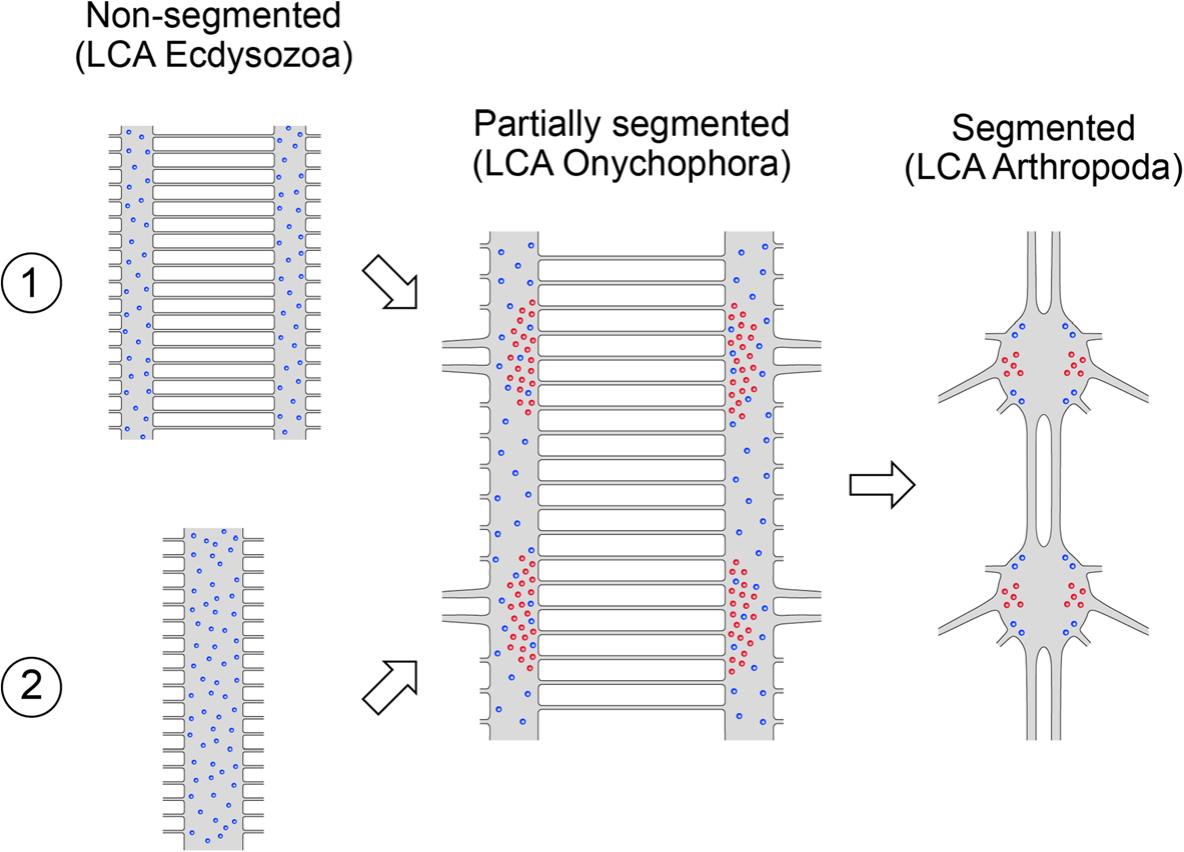
Scenario of the evolution of segmentation in the ventral nerve cord within ecdysozoans. Simplified diagrams of the ventral nerve cords of putative last common ancestors of Ecdysozoa (*left*), Onychophora (*middle*) and Arthropoda (*right*). Note the two alternative hypotheses regarding the architecture of the ventral nervous system in the last common ancestor of ecdysozoans with either a paired ventral nerve cord (*1*) or a median unpaired ventral nerve cord (*2*; modified from Yang et al. [7]). Note also the stepwise increase in the degree of segmentation of the ventral nervous system. Diagrams of the onychophoran and arthropod nervous systems according to Mayer and Harzsch [11] and Richter et al. [39], respectively. Neuronal cell bodies of the ventral nerve cord are indicated in blue and somata of motor neurons of leg nerves are indicated in red. LCA, last common ancestor

To suggest that tardigrades form the sister group to all remaining panarthropods, one would have to assume that segmental ganglia are indeed an ancestral feature that experienced a modification within the onychophoran lineage, in that the ganglionic cell bodies were re-distributed along the whole nerve cords, thus resulting in their medullary organisation. Alternatively, if tardigrades and arthropods form the sister group to onychophorans, one would suggest that medullary ventral nerve cords, with local accumulations of motor and presumably other neurons at the base of each leg, as still seen in onychophorans, had already been present in the panarthropod ancestor. Therefore, this partly segmentally arranged nerve cord might have been a prerequisite for the evolution of ganglia in the tardigrade and arthropod lineage, suggesting that the segmental ganglia evolved by a gradual condensation of neuronal subsets. Our data show that an accumulation of neuronal cell bodies within specific regions of the nerve cord most likely correlates with either the presence of leg nerves or sets of musculature within the legs. This is in line with the assumption that segmentation evolves gradually through individual, repeated structures [35–37] rather than as complete holistic units [33, 34]. Thus, we suggest that the onychophoran nerve cords show an ancestral arrangement of the ventral nerve cord (similar to the presumable ventral nervous system of the last common ancestor in panarthropods) — albeit with several segmental features already present — whereas the segmental ganglia most likely evolved in the tardigrade-arthropod lineage (Fig. 11). Accordingly, our data do not support previous assumptions that segmentation was already present in the last common ancestor of all protostomes ([34, 62, 63] but see [13, 28, 35, 64, 65] for a different view). Assuming that segmentation evolved only once would consequently imply secondary losses in many other animal groups, such as Platyhelminthes, Mollusca or Cycloneuralia.

Within arthropods, two different types of segmentation are known: (i) the initial metameric units, the parasegments, that are present during embryonic development and (ii) the segments as seen in adults [66, 67]. Within adults, the presence of parasegments is represented by an anteriorly shifted position of ganglia with respect to the borders of true segments [66]. Previously, the presence of both segmentation types was suggested to be either an autapomorphy of all panarthropods [63] or a synapomorphy of tardigrades and arthropods [3]. So far, only the shifted position of ganglia [3, 68] indicates the presence of parasegments in tardigrades, as corresponding gene expression data are lacking. Nevertheless, whether or not onychophorans share none, one or both of these segmentation types with arthropods (and tardigrades) is still under debate [28, 69]. Although the existence of medullary nerve cords in onychophorans precludes the identification of parasegments based on position alone, recent expression data from segment polarity genes [28] suggest that parasegments, if present at all, do not form the initial metameric unit (contrary to [63]) within the onychophoran embryo as known from arthropods [66, 67]. Additionally, our data show that, regardless of the marker used, an anterior shift of an accumulation of neuronal cell bodies with respect to the legs is not present within the onychophoran nerve cords. Thus, based on evidence from both developmental and immunohistochemical data, we suggest that parasegments were not present within the last common ancestor of panarthropods but instead are either an autapomorphy of arthropods or evolved in the last common ancestor of tardigrades and arthropods. Alternatively, if tardigrades represent the basal-most group within the panarthropods, one would assume a loss of parasegments in onychophorans.

Unfortunately, the neuroanatomical characteristics of the last common ancestor of Ecdysozoa are still unknown. Currently, there are two alternative hypotheses regarding the layout of the ventral nerve cords within the last common ancestor (LCA) of ecdysozoans ([7, 13, 70, 71]; both left diagrams in Fig. 11). Yang et al. [7] hypothesised that the ancestral ecdysozoan possessed an unpaired ventral nerve cord (bottom left diagram in Fig. 11), which was retained in most cycloneuralians, a subclade of ecdysozoan animals [72]. Therefore, a separation of the ventral nerve cord would have occurred in the LCA of panarthropods, as all three panarthropod subgroups show paired ventral nerve cords [3, 11, 50], as well as independently within the cycloneuralian subgroup Loricifera [73, 74]. An alternative hypothesis suggests paired ventral nerve cords to be the ancestral feature of all ecdysozoans ([13]; upper left diagram in Fig. 11), thus leading to the assumption that a fused unpaired ventral nerve cord constitutes an autapomorphy of cycloneuralians. Furthermore, most subgroups of the Spiralia (which form the sister group to the Ecdysozoa [75, 76]) exhibit paired neural tracts (e.g. Gnathostomulida, Gastrotricha, Platyhelminthes, Mollusca, Nemertea [70, 77]) with few exceptions, such as the highly diverse Annelida. Instead, annelids show a considerable degree of variation regarding their ventral nervous systems [78] but the ground pattern is potentially trineuralian (a pair of lateral nerve cords and an unpaired median nerve cord [79]), with varying degrees of fusion among the annelid subgroups [78]. In any case, Hejnol and Lowe [70] note that the ventral nervous system was most likely centralised independently in arthropods and annelids. Therefore, the ancestral condition of the ventral nerve cords within Ecdysozoa (paired versus unpaired) remains ambiguous and requires further investigation.

Most recently, Yang et al. [7] investigated a well-preserved ventral nervous system of the more than 500 million-year-old stem-group arthropod fuxianhuiid fossil †*Chengjiangocaris kunmingensis* (Cambrian Series 2, Stage 3). Based on their findings, the authors suggest that the complex, ganglionated ventral nervous system in tardigrades and arthropods evolved within their common lineage, whereas the onychophoran nerve cord shows the ancestral state within panarthropods. Although some previous authors described a ganglionated nerve cord in the lobopodian fossil †*Paucipodia inermis* [80], whether or not this interpretation is valid is still under vigorous debate [7, 81]. Thus, the latest study of Yang and colleagues [7] coincides with our findings, which suggest a stepwise evolution of the segmented nervous system within panarthropods (Fig. 11) rather than a loss of an ancestral segmentation within the onychophoran lineage.

## Conclusions

To date, the organisation of the nervous system and particularly its evolution within panarthropods remains an enigmatic feature causing various contentious presumptions, many of which surround the issue of segmentation of the nervous system within all three panarthropod groups [3, 7, 9, 11–13, 21, 34, 35, 38, 40, 41]. Our findings, based on neuroanatomical investigations in onychophorans, clearly revealed the presence of both segmental and non-segmental features within their ventral nervous system. This pattern shows a notable correspondence in position between structures such as musculature and peripheral nerves, thus leading to the assumption that the arrangement of the neurons in the onychophoran nervous system is dictated solely by the organisation of the structures they supply instead of being organised in functional units (ganglia) as known from arthropods. Therefore, our data contradict previous assumptions of ganglia being already present in the last common ancestor of panarthropods and being reduced in onychophorans [9, 44, 46]. Consequently, we suggest that the segmental ganglia of arthropods evolved by a gradual condensation of subsets of neurons within the arthropod or arthropod-tardigrade lineage and with onychophorans representing the ancestral state within panarthropods. These findings are in line with the hypothesis of a gradual evolution of segmentation within panarthropods [28, 35–37].

## Methods

### Specimen collection and maintenance

Specimens of *Euperipatoides rowelli* Reid, 1996 (Onychophora, Peripatopsidae) were collected from decaying logs in the Tallaganda State Forest (New South Wales, Australia; 35°26’S, 149°33’E, 954 m) in January 2013. Permission for specimen collection was obtained from the Forestry Commission of New South Wales (permit no. SPPR0008). Export permission was issued from the Department of Sustainability, Environment, Water, Population and Communities (permit no. WT2012-8163). Specimens of *Principapillatus hitoyensis* Oliveira et al., 2012 (Onychophora, Peripatidae) were obtained in October 2005 in the Reserva Biológica Hitoy Cerere (Province of Limón, region of Talamanca, Costa Rica, 09°40’21”N, 83°02’36”W, 300 m). Federal permission for specimen collection was provided via the resolution number 123-2005-SINAC and exported under the federal permission number 014950 provided by the Gerencia Manejo y Uso Sostenible de RR NN – Ministerio del Ambiente y Energía. Living specimens of both species studied were maintained in plastic boxes with perforated lids as described previously [31, 82]. The experiments in this study did not require an approval by an ethics committee. All procedures in this investigation complied with international and institutional guidelines, including the guidelines for animal welfare as laid down by the German Research Foundation (DFG). We have chosen the model onychophoran *E. rowelli* as the focus of our study because many aspects of its biology, including anatomy, development, reproduction, and phylogeny, have been investigated extensively [83].

### Retrograde fills

For neuronal staining, adult specimens were anaesthetised in chloroform vapour for 10–20 s and cut open longitudinally along the dorsal side using fine scissors as described previously [18]. The ventral nerve cords were dissected in physiological saline [84] and pinned down with tungsten needles in small Petri dishes coated with Sylgard® (184 Silicone Elastomer Kit, DowCorning GmbH, Wiesbaden, Germany). Individual leg nerves and median- and ring commissures were dissected from surrounding tissue and a well of Vaseline was built around each isolated nerve, after which the saline was removed from the well and replaced with distilled water. A few crystals of dextran coupled to either tetramethylrhodamine or fluorescein (MW3000, lysine-fixable; Molecular Probes, Eugene, USA) were then added to the well filled with distilled water [85]. The preparations were incubated in the dark for 12–16 h at 4 °C, after which the well containing dextran was removed and the tissue fixed in 4% paraformaldehyde (PFA) in 0.1 M phosphate-buffered saline (PBS; pH = 7.4) for 2 h at 4 °C. After several rinses in PBS and dehydration through an ethanol series (50, 70, 90, 95%, 2 × 100%; 10 min each) the preparations were cleared and mounted in methyl salicylate between two coverslips.

### Immunohistochemistry

To gain a representative impression of the organisation of the onychophoran nervous system we used a variety of established antisera directed against well-known neuronal signaling molecules (serotonin, gamma-aminobutyric acid [GABA], dopamine, octopamine, tyramine, allatostatin, pigment-dispersing factor [PDF, referred to as pigment-dispersing hormone in crustaceans], perisulfakinin, and RFamide) that are common in arthropods, along with markers for nerve fibres (acetylated α-tubulin), a synaptic protein (synapsin) and cell nuclei (DNA). Each of the employed antisera has been shown to be specific and selective for its target molecule in a variety of arthropods (details below). However, since the specificity of the antisera has not been specifically tested on onychophoran tissue, we refer to labelled structures as expressing antigen “-like immunoreactivity”. For each antiserum we performed the standard controls of omitting either the primary or secondary antiserum in the staining protocol, and this resulted in no staining of nerve cell bodies or processes thereof in all cases.

#### Anti-acetylated α-tubulin immunohistochemistry

The anti-α-tubulin serum was raised in mouse against acetylated α-tubulin from the outer arm of the sea urchin *Strongylocentrotus purpuratus* (Sigma-Aldrich® Co., St. Louis, USA). The monoclonal antibody recognises an epitope located on the α3 isoform of *Chlamydomonas* axonemal α-tubulin [86], within four residues of Lys-40 when this amino acid is acetylated [87]. Specimens were anaesthetised in chloroform vapour and fixed in 4% PFA in PBS at room temperature. After several rinses in PBS specimens were embedded in 7% agarose gel at 60 °C and cooled to room temperature. The blocks were then trimmed and sectioned into 60–80 μm thin sections with steel blades on a Vibratome (V 1000 plus Sectioning System, Vibratome Company). The sections were washed with several rinses of PBS containing 1% Triton X-100 (PBS-Tx, Sigma-Aldrich® Co., St. Louis, USA) and then incubated in PBS-Tx containing 1% bovine serum albumin (Sigma-Aldrich® Co., St. Louis, USA). After incubation with the primary antibody (diluted 1:500 in PBS containing 1% normal goat serum [NGS; Sigma-Aldrich® Co., St. Louis, USA]) overnight at 4 °C, sections were washed in several rinses with PBS-Tx. Thereafter, sections were incubated in the secondary antibody (goat anti-mouse Alexa Fluor® 488 LifeTechnologies™, Molecular Probes®, Carlsbad, USA, 1:500 in PBS) overnight at 4 °C. After several rinses in PBS, sections were mounted on glass slides in Vectashield® Antifade Mounting Medium (Vector Laboratories Inc., Burlingame, CA, USA).

#### Anti-synapsin immunohistochemistry

The monoclonal anti-synapsin serum (antiSYNORF1; [88]) was provided by Dr Bert R. E. Klagges (University of Leipzig, Germany). This antibody is a marker of synaptic terminals and was raised in mouse against the synapsins of *Drosophila melanogaster* (see [88]). The staining protocol is based on that of Ott [89] developed initially for locusts. The nerve cords were dissected in HEPES-buffered saline (HBS: 10 mM HEPES, 25 mM sucrose, 150 mM NaCl, 5 mM KCl, 5 mM CaCl_2,_ pH 7.4), pinned down in small Petri dishes coated with Sylgard® and fixed in zinc-formaldehyde (ZnFA: 18.4 mM ZnCl_2_, 135 mM NaCl, 35 mM sucrose, 1% formaldehyde) for 20 h. After several rinses in HBS, the nerve cords were dehydrated in an 80% methanol and 20% dimethyl sulfoxide (DMSO) mixture for 1.5 h at room temperature and subsequently transferred to 100% methanol for 1 h. After dehydration the nerve cords were rehydrated through a methanol series (90, 70, 70, 30% in Tris-buffered saline [TBS; 50 mM Tris, 150 mM NaCl, pH 7.4], 7 min each) and then blocked in a solution containing TBS with 5% normal goat serum and 1% DMSO for 1 h. All subsequent steps were carried out in the dark. Subsequently, nerve cords were incubated with the primary antibody (diluted 1:50 in TBS, 5% NGS, 1% DMSO) for 3.5 days at 4 °C. Thereafter, the nerve cords were washed several times in TBS with 1% DMSO (TBS-D) and incubated with the secondary antibody (goat anti-mouse Alexa Fluor® 568 LifeTechnologies^TM^, Molecular Probes®, Carlsbad, USA, 1:500 in TBS) for 2.5 days, after which they were washed in TBS-D, dehydrated through an ethanol series (70, 90, 95, 100, 100%, 10 min each), cleared, and mounted between two coverslips in methyl salicylate. We refer to the labelled structures in our specimens as expressing “synapsin-like” immunoreactivity.

#### Anti-gamma-aminobutyric acid immunohistochemistry

The anti-GABA serum was obtained by immunising rabbits with GABA-glutaraldehyde-bovine serum albumin (BSA)-conjugate [90, 91]. The non-proteinogenic gamma-aminobutyric acid (GABA) antiserum was obtained from Dr Manfred Eckert (Friedrich Schiller University, Jena, Germany), who used it to demonstrate its specifity in insects [92]. The nerve cords were dissected in ice-cold physiological saline [84] and fixed in 4% glutaraldehyde containing 15% picric acid and 0.5% glacial acetic acid in PBS for 3 h at 4 °C, after which they were washed several times in PBS, dehydrated through an ascending ethanol series (70, 90, 95, 100 and 100%, 10 min each), cleared in xylene, rehydrated through a descending ethanol series (100, 100, 95, 90, 70 and 50%, 5 min each) and washed again several times in PBS. The nerve cords were then incubated in sodium borohydride (1% in PBS) for 30 min to reduce autofluorescence due to glutaraldehyde fixation. After several rinses in PBS, the nerve cords were treated with a mixture of collagenase/dispase (Roche Diagnostics GmbH, Mannheim, Germany; each 1 mg/mL) and hyaluronidase (Sigma-Aldrich® Co.; 1 mg/mL) in PBS for 40 min at 37 °C. After several washes in PBS-Tx, the nerve cords were incubated in 10% NGS in PBS-Tx for 1.5 h, followed by an incubation with the primary antibody (diluted 1:500 in PBS-Tx, 1% NGS) for 3 days at 4 °C. After several rinses in PBS-Tx, the nerve cords were incubated with the secondary antibody (goat anti-rabbit Alexa Fluor® 568, LifeTechnologies™, 1:500) for 24 h at 4 °C. The specimens were then washed in PBS-Tx, dehydrated through an ethanol series, cleared in methyl salicylate and mounted between two coverslips. We refer to the labelled structures in our specimens as expressing “GABA-like” immunoreactivity.

#### Anti-dopamine immunohistochemistry

The polyclonal anti-dopamine serum was obtained from Dr Harry W. M. Steinbusch (Maastricht University, Netherlands). It was raised in rabbit against dopamine coupled to thyroglobulin by means of glutaraldehyde. Its specificity has been tested by Steinbusch and Tilders [93] and Steinbusch et al. [94]. The staining protocol is based on that of Stevenson et al. [95], developed for insects. The nerve cords were dissected in ice-cold sodium cacodylate buffer (pH 6.2) and fixed in 6% glutaraldehyde in 0.1 M sodium cacodylate buffer containing 0.5% glacial acetic acid with 1% sodium metabisulphite (SMB) for 3 h at room temperature. Fixation and all subsequent steps including the incubation with the primary antibody were performed in the dark and all solutions contained 1% SMB. After fixation, the nerve cords were washed several times in PBS containing 1% sodium borohydride. The subsequent enzyme treatment and washing steps were the same as for the anti-GABA immunohistochemistry. The nerve cords were incubated with the primary antibody (diluted 1:1000) for 3 days at 4 °C. All subsequent steps were the same as in the anti-GABA protocol. We refer to the labelled structures in our specimens as expressing “dopamine-like” immunoreactivity.

#### Anti-serotonin immunohistochemistry

The polyclonal antiserum was raised in rabbit against the biogenic amine serotonin coupled to *Limulus polyphemus* haemocyanin (NT-102 Eugene Tech Inc., NJ, USA; currently Protos Biotech, NJ, USA). This antiserum was shown to recognise serotonergic neurons in molluscs [96], insects [95] and rats [97]. The dissected nerve cords were fixed in 3% PFA in PBS and treated as described above for the anti-GABA immunohistochemistry, except for the following modifications: The nerve cords were not incubated in sodium borohydride, which is only required for the glutaraldehyde fixation, and the primary antibody was diluted 1:1500. We refer to the labelled structures in our specimens as expressing “serotonin-like” immunoreactivity.

#### Anti-octopamine immunohistochemistry

The polyclonal antisera were raised in rabbits after immunisation with octopamine-glutaraldehyde-BSA-conjugate (MoBiTec, Göttingen, Germany). Its specificity has been tested with an indirect competitive-enzyme-linked immunosorbent assay (ELISA) [98] and revealed a high affinity to the tested octopamine-glutaraldehyde-BSA conjugate. A relative cross-reactivity has been tested to noradrenaline-glutaraldehyde-BSA (1:90), tyramine-glutaraldehyde-BSA (1:142) and _L_-3,4-Dihydroxyphenylalanine (L-DOPA)-glutaraldehyde-BSA (1:285), dopamine-glutaraldehyde-BSA (1:1120), adrenaline-glutaraldehyde-BSA (1:>10,000) and unbound octopamine (1:>10,000). All steps were carried out in the dark. The nerve cords were dissected in ice-cold physiological saline [84] and fixed in 1% glutaraldehyde containing 15% picric acid and 0.5% glacial acetic acid in PBS for 2 h at room temperature. The primary antibody was diluted 1:500 and detected using goat anti-mouse Alexa Fluor® 568 (LifeTechnologies^TM^, 1:500). The staining protocol was otherwise as for the anti-GABA immunohistochemistry. We refer to the labelled structures in our specimens as expressing “octopamine-like” immunoreactivity.

#### Anti-tyramine immunohistochemistry

The polyclonal antiserum (Chemicon International, Temecula, CA) was raised in rabbit against p-tyramine-glutaraldehyde-N-α-acetyl-L-lysine-N-methylamide. Cross reactivity (determined using either ELISA or radioimmunoassay) was detected for conjugates of octopamine-glutaraldehyde-BSA (0.125%), dopamine-glutaraldehyde-BSA (<0.0025%) and tyrosine-glutaraldehyde-BSA (<0.002%) [99]. All steps were carried out in the dark and all solutions up to the primary antibody contained 1% SMB. The nerve cords were dissected in ice-cold physiological saline [84] containing 1% SMB and fixed in 6% glutaraldehyde containing 15% picric acid and 0.5% glacial acetic acid in PBS for 2 h at room temperature. The staining protocol was otherwise as for the anti-GABA immunohistochemistry, except for the following modifications: the nerve cords were incubated for 5.5 days with the primary antibody (diluted 1:500) and the primary antibody was detected using goat anti-rabbit-Cy™3 (Dianova, Hamburg, Germany; diluted 1:200). We refer to the labelled structures in our specimens as expressing “tyramine-like” immunoreactivity.

#### Anti-allatostatin immunohistochemistry

The polyclonal anti-allatostatin serum was obtained from Dr Hans-Jürgen Agricola (Friedrich Schiller University, Jena, Germany). It was raised in rabbit against the peptide allatostatin I (Dip-allatostatin I, APSGAQRLYGFGL-amide) from the cockroach *Diploptera punctata*. The antiserum recognises allatostatins I–V, but is almost two orders of magnitude more sensitive to AST-1 than to the other ASTs [100]. The nerve cords were dissected as described for the anti-GABA immunohistochemistry and fixed in 4% PFA in PBS. The staining protocol was otherwise as for the anti-serotonin immunolabelling. The primary antibody was diluted 1:5000. We refer to the labelled structures in our specimens as expressing “allatostatin-like” immunoreactivity.

#### Anti-pigment-dispersing factor/hormone immunohistochemistry

The polyclonal anti-pigment-dispersing hormone antibody was provided by Dr Heinrich Dircksen. It was raised in rabbit against synthetic β-PDH of *Uca pugilator* [48]. The nerve cords were dissected as described for the anti-GABA immunohistochemistry and fixed overnight at room temperature in 2% paraformaldehyde containing 15% picric acid in 0.1 M PBS [101]. The staining was performed as described previously [19]. We refer to the labelled structures in our specimens as expressing ““PDF-like” (pigment-dispersing factor-like) immunoreactivity.

#### Anti-perisulfakinin immunohistochemistry

The antiserum against pea-sulfakinin (perisulfakinin) was raised in rabbit and provided by Dr Hans-Jürgen Agricola (Friedrich Schiller University, Jena, Germany). The antiserum was tested in various insects, spiders, crustaceans, centipedes and annelids [102, 103]. Perisulfakinin belongs to a family of peptides with identical C-terminal sequences (sulfakinins) that share structural similarities with vertebrate cholecystokinin/gastrin peptide and invertebrate FMRFamide-like peptides [102, 103]. The nerve cords were treated as described for the anti-allatostatin immunohistochemistry. The primary antibody was diluted 1:1000. We refer to the labelled structures in our specimens as expressing “perisulfakinin-like” immunoreactivity.

#### Anti-FMRFamide immunohistochemistry

The polyclonal antiserum (Incstar, Stillwater, USA; currently ImmunoStar, Hudson, WI, USA) was raised in rabbit against the neuropeptide FMRFamide coupled to bovine thyroglobulin. According to the company, the FMRFamide antiserum was quality control tested using standard immunohistochemical methods. The antiserum has been tested and used in various arthropods (e.g. locusts; [104]) The nerve cords were dissected in ice-cold physiological saline [84] and otherwise treated as described for the anti-serotonin immunohistochemistry. The primary antibody was diluted 1:2000. We refer to the labelled structures in our specimens as expressing “RFamide-like” immunoreactivity since the antibody labels a variety of peptides terminating with the sequence RFamide.

#### DNA labelling

The DNA-selective fluorescent dyes SYBR® Green (Invitrogen, Carlsbad, CA, USA; 1:10,000 in PBS) or Hoechst (Bisbenzimide, H33258, Sigma-Aldrich; 1 μg/ml in PBS) were applied for counterstaining according to the manufacturers’ protocols.

### Confocal microscopy, light microscopy and image processing

Whole-mount preparations of the ventral nerve cords were analysed with epifluorescence (Leica Leitz DMR; Leica Microsystems, Wetzlar, Germany) and confocal laser-scanning microscopes (CLSM, Leica TCS STED; Leica Microsystems). Confocal image stacks were processed with Leica ASAF v2.3.5 (Leica Microsystems), IMARIS 7.2.1 (Bitplane, Zürich, Switzerland), and ImageJ 1.49m ([105], National Institutes of Health, Bethesda, MD, USA). Final panels and diagrams were designed using Adobe (San Jose, CA, USA) Photoshop CS5 and Illustrator CS5.

### Cell counts

Cell bodies were stained with each specific marker and then quantified per hemisegment using whole CLSM image stacks. A hemisegment was defined as the region of one nerve cord delimited by two consecutive anterior/posterior leg nerves. Each hemisegment was subdivided into ten equal portions, in which labelled cell bodies of each marker were counted by using Fiji ImageJ 1.49v [106]. Each marker was only analysed in one nerve cord due to limited material. The χ^2^-test from a standard statistics package (Prism 6.0, Graphpad Software Inc. La Jolla, CA, USA) was employed to evaluate whether the actual distribution of stained somata in the ten portions of a hemisegment differed significantly from a theoretically expected uniform distribution with no regional specialisation in the nerve cord. The number of nuclei stained with the DNA-selective fluorescent dye DAPI (1 μg/ml; Carl Roth, Karlsruhe, Germany) was estimated by using sections (20 μm thickness) of a dissected nerve cord from one body side created with a cryostat (Leica CM3050 S, Leica Biosystems, Wetzlar, Germany). For comparing numbers of somata within the sections of the leg-bearing and the interpedal region of the nerve cord a two tailed Student’s *t*-test for unpaired data sets was used.

## Additional file

Additional file 1: Figure S1. Light micrographs of cross sections, stained with a DNA-selective marker, to visualise differential circumferences of the nerve cord within the interpedal and the leg-bearing regions in Onychophora. (PDF 114 kb)
