# Supplementary material for: Assessing segmental versus non-segmental features in the ventral nervous system of onychophorans (velvet worms)
Source: BMC Evol Biol. 2017 Jan 3;17:3. doi: 10.1186/s12862-016-0853-3 (PMC5209844; doi:10.1186/s12862-016-0853-3)
Supplement: Additional file 1: Figure S1. — Light micrographs of cross sections, stained with a DNA-selective marker, to visualise differential circumferences of the nerve cord within the interpedal and the leg-bearing regions in Onychophora. (PDF 114 kb) [file 12862_2016_853_MOESM1_ESM.pdf]

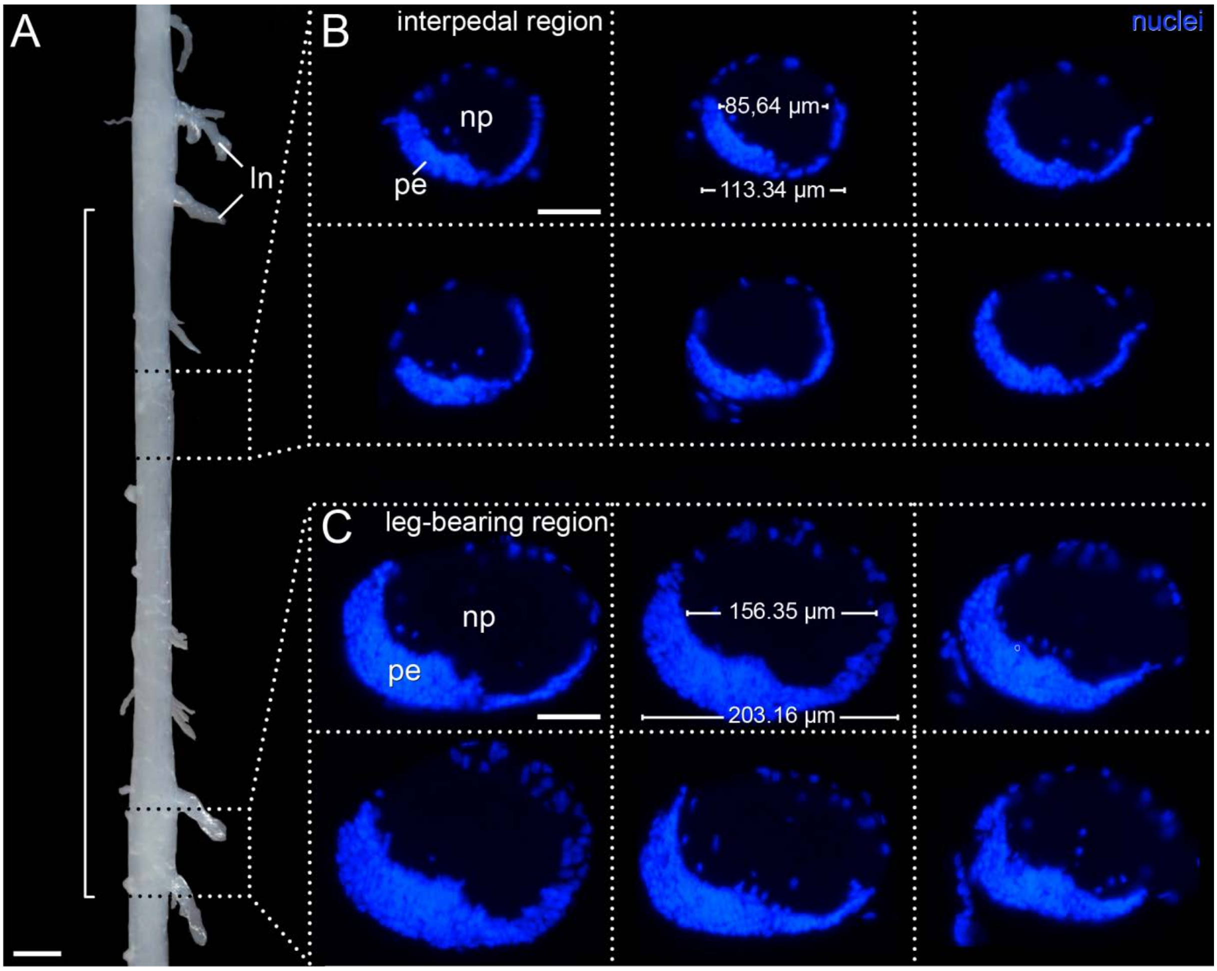

Supplementary Figure 1 Differential circumferences of the nerve cord within the interpedal and the leg-bearing regions in Onychophora. Light micrographs **(a)** and fluorescence micrographs **(b, c)**. Median is left in all images, dorsal is up **(b, c)**. **a** Dissected nerve cord of a juvenile *Euperipatoides rowelli*. *Bracket* indicates one hemisegment from one body side delimited by two consecutive posterior leg nerves. *Dotted lines* indicate the region of the sections represented as insets **(b, c)**. **b, c** Cross sections of the interpedal region **(b)** and the leg-bearing region **(c)** of the same nerve cord as in **a** stained with a DNA-selective marker (DAPI). All sections are to scale. Note the differences in circumferences and number of nuclei comparing the interpedal and the leg-bearing regions. Additionally, the region of the neuropil is more voluminous in the leg-bearing region. ln, leg nerve; np, region of the neuropil; pe, perikaryal layer. Scale bars: 200 µm **(a)** and 50 µm **(b, c)**
